# Assessment of gait in mice using simplified analysis tools

**DOI:** 10.21203/rs.3.pex-2191/v1

**Published:** 2023-05-05

**Authors:** Raghda Fouda, Donovan Argueta, Kendall O'Daniel, Kristen Peterson, Tiffany Sorto, Kalpna Gupta

**Affiliations:** Department of Medicine, Division of Hematology/Oncology, University of California, Irvine, CA, United States; Department of Medicine, Division of Hematology/Oncology, University of California, Irvine, CA, United States; Department of Medicine, Division of Hematology/Oncology, University of California, Irvine, CA, United States; Department of Medicine, Division of Hematology/Oncology, University of California, Irvine, CA, United States; Department of Medicine, Division of Hematology/Oncology, University of California, Irvine, CA, United States; Department of Medicine, Division of Hematology/Oncology, University of California, Irvine, CA, United States. Department of Medicine, Division of Hematology, Oncology and Transplantation, University of Minnesota, Minneapolis, MN, United States. Southern California Institute for Research and Education, Veterans Affairs (VA) Medical Center, Long Beach, CA, United States

**Keywords:** Gait analysis, pain, hemophilia, MouseWalker, weight-bearing, arthropathy, mouse model

## Abstract

Gait analysis has received significant attention in many clinical conditions including chemotherapy-induced alterations, degenerative diseases, and hemophilia. Gait changes can be a consequence of physical and/or neural/motor alterations and/or pain. It can provide measurable objective outcomes for following disease progression and the effectiveness of therapy without patient or observer bias. Many devices are available for analyzing gait in clinic. Gait analysis in laboratory mice is frequently used to examine the mechanisms and effectiveness of interventions for movement and pain assessment. However, gait analysis in mice is challenging due to the complexity of image acquisition and analysis of large data sets. We have developed a relatively simple method to analyze gait and validated it using the arthropathy model in hemophilia A mice. We describe artificial intelligence-assisted detection of gait and validation with weight-bearing incapacitance for stance stability in mice. These approaches enable the non-invasive, non-evoked evaluation of pain and the consequent impact of motor function on gait.

## Introduction

The MouseWalker software is an open-source program that is easily accessible, but we faced technical challenges initiating and operating the software using the standalone executable program file. However, we were able to successfully deploy the program by opening the source code using MATLAB, which allowed for seamless video analysis and exportation of datasheets (detailed list of software requirements provided in Materials).

Pain in hemophilia, a rare blood disorder caused primarily due to arthropathy, remains under investigation. Characterized by bleeding in the joints, hemophilia affects mobility in addition to chronic and acute pain. Currently, the most used pain assessment methods in people with hemophilia are visual or numerical rating scales, pain questionnaires ^1^, and quality-of-life questionnaires ^2^. However, an objective quantitative method for pain detection is lacking, which precludes accurate assessment of pain in juvenile hemophilia. Pain has been related to movement instabilities in arthritic disorders ^3^. Thus, we hypothesized that pain would impair the gait and stance stability parameters in a mouse model of hemophilia A.

Gait analysis with the MouseWalker system is a non-invasive method for detecting subtle changes in rodent movement behaviors, which may underly sensory, inflammatory, and neurological pathobiology ^4^. This method has technical challenges that impose a barrier to its utility. Thus, this protocol aims to make MouseWalker gait assessment accessible and simple for most murine behavioral laboratories.

Our group has successfully implemented the MouseWalker workflow to perform gait analysis in a mouse model of sickle cell disease (SCD) ^5^. Gait and movement parameters may provide objective, meaningful data for the study of pain in people with hemophilia, which is supported by several studies indicating the contribution of pain to changes in gait ^6^. Weight-bearing is a complementary test of non-evoked pain and stance instability that has been used to evaluate pain in studies of arthritic conditions ^7^. Early identification of gait changes may provide clinicians with the opportunity to intervene with the aim of arresting the progression of joint damage. Thus, we hypothesized that pain would alter weight bearing and gait in a factor-VIII knockout mouse model of hemophilia A.

## Reagents

- Wild-Type B6.129SF2/J (#101045, Jackson Laboratories, Bar Harbor, ME, USA) control mice
- Factor VIII knockout B6. 129S-F8^tm1Kaz^/J (#00444, Jackson)
- 70 % ethanol (#BP2818-4, Thermo Fisher Scientific, Waltham, MA, USA)

## Equipment

**Figure 1:**
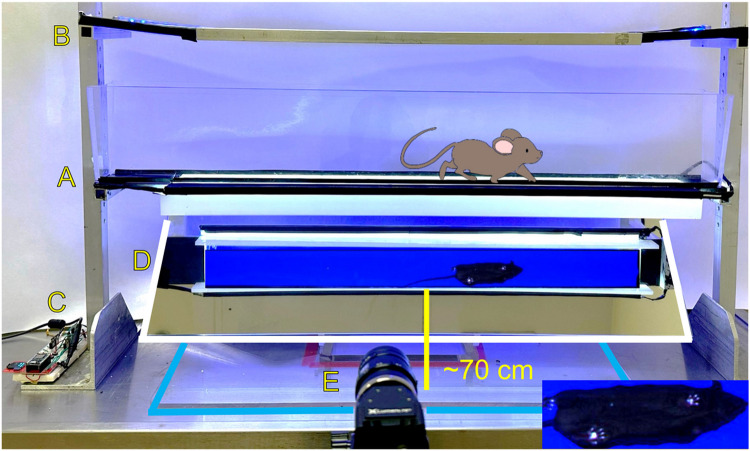
MouseWalker apparatus. (**A**) The mouse walks freely down a plexiglass walkway (8 × 80 cm2) with embedded white LEDs and side barriers, and light propagates within the glass via internal reflection, i.e., *total internal reflection* (TIR). During the mouse’s natural walking gait, only in those areas where the mouse’s paws make contact with the glass plate is the light scattered, causing *frustrated total internal reflection* (fTIR) within the transparent material. (**B**) Constant background lighting is established in the backlight panel with embedded, adjustable-colored LEDs (blue in the illustration) placed 40 cm over the walkway. The background light allows the outline of the mouse’s body to be visualized as the animal moves along the walkway. (**C**) A programmable controller adjusts light intensity for LEDs. (**D**) Shows the actual acquisition of the body and paws of a mouse on the walkway. A polished mirror (outlined in white) and stand maintained at a 45° angle beneath the walkway reflects the fTIR signal and body outline, allowing them to be captured by (**E**) a high-speed camera CMOS camera at approximately 70 cm distance from the mirror. Inset magnifies the mouse paw prints as they appear in the video recordings.

- Ugo Basile Incapacitance Tester (#54783, Stoelting Co., Wood Dale, IL, USA), Weight Bearing Device
- MouseWalker apparatus was custom-built using campus machine shop for metal components, low-reflection clear acrylic, and a mirror for video capture (see Figure 1).
- High-Speed CMOS Camera (#Lt425C, Lumenera Corporation, Ottawa, Ontario)
- LEDs for background and total internal reflection (TIR; #HL-LS5050_RGB300NW44K, HitLights, Chino, CA, USA; #9001-K25, MacMaster-Carr, Elmhurst, IL, USA)
- Laptop Computer with Windows 8.1 (or newer), minimum 8 GB memory, USB 3.0 (or newer) x 2, and minimum 256 GB storage
- Generic USB 3.0 Cable.

### Software

- MATLAB v.9.11 or newer (The Mathworks, Natick, MA, USA) with the following libraries installed:
- Curve Fitting Toolbox
- Econometrics Toolbox
- Sensor Fusion and Tracking Toolbox
- GPU Coder
- Image Acquisition toolbox
- Image Processing Toolbox
- MATLAB Coder
- Parallel Computing Toolbox
- StreamPix6 or newer (NorPix, Montreal, Canada)
- ImageJ (National Institutes of Health, Bethesda, MD, USA)
- Prism6 or newer (GraphPad, New York, NY, USA

## Procedure

The data is in the form of representative figures and an excel data sheet for all the assessed parameters.

### A. Weight Bearing (Approximate Time: 120 minutes)

1. Acclimatize mice in the behavioral testing room approximately 15-20 minutes prior to testing. It is recommended to expose mice to the testing environment for at least 2 days prior to experimentation.

2. Clean surfaces of testing chambers and equipment with 70% ethanol and allow surfaces to dry completely before placing mice.

3. The device should be placed and maintained on a flat, sturdy surface or bench top. Installation and power requirements are described in more detail by the manufacturer (https://stoeltingco.com/Neuroscience/Ugo-Basile-Incapacitance-Tester~10547, accessed 12/12/2022).

4. Connect the device to an appropriate power outlet and initiate using the power button.

5. Adjust the duration of recording to the desired length (in seconds); presently, we recorded for a period of 5 seconds in triplicate.

6. Press “Start” on the screen.

7. Press “zero” to tare the pressure-sensitive plates prior to calibration (with dedicated, standard weights) or data acquisition.

8. Place mice into the testing chamber, oriented with hind paws on pressure-sensitive plates and fore paws on elevated interior surface. Allow the mouse to explore the chamber until it has stopped for >5 s intervals.

9. Reorient the mouse onto pressure-sensitive plates with each hind paw evenly placed, then initiate recording.

10. The weight-bearing capacity of each hind paw is automatically measured and averaged, then the device display will indicate the final value for each paw to the nearest 0.1 g.

11. Record these values separately, then repeat to obtain triplicate values for each subject.

12. Calculate stance instability by determining the absolute (>0) difference between left and right hind paw weight bearing as a percentage of total weight-bearing capacity (sum of left and right recordings).

### B. Gait analysis

#### B.1 Calibration Procedure (Approximate Time: 5 minutes)

1. To appropriately calibrate MouseWalker software for quantitative gait analysis, the scale for pixel size (pixels/cm) must be determined empirically.

2. Make a demarcation of fixed length (e.g., 2 cm) or place a translucent metric ruler on the surface of the MouseWalker walkway, matching the side of the mouse placement.

3. Capture reference length with either video or still image capture, while preserving camera location and position for subsequent recordings.

4. Using ImageJ (NIH) software, open the video or image file with the ruler or marking. Within the *“Analyze”* drop-down toolbar, select *“Set Scale”* and input the known distance and unit in cm to calculate the pixel per cm parameter.

5. Save this parameter for the MouseWalker software setup.

#### B.2 Recording procedures (Approximate Time: 60 minutes)

1. Acclimatize the mice in a darkened testing room 15 minutes before starting the experiment. Appropriate acclimatization is crucial, especially given that low light is required for proper light detection. It is recommended to expose mice to the testing environment for at least 2 days prior to experimentation.

2. Prepare the MouseWalker apparatus for recording. All MouseWalker surfaces are thoroughly disinfected between use, and acrylic surfaces for light interference recording are cleaned with 70% ethanol to maintain clarity and remove any debris/dust. Allow the surfaces to dry completely before starting the experiment.

3. Initiate a light controller for light interference (white light) in the acrylic walkway and background light. Set the background light color to *“blue”* at the highest light intensity, however light intensity and color should be determined empirically.

4. Video is acquired using a high-speed CMOS camera (Lumenera) connected to the central workstation via a USB 3.0 connection. Set camera position, iris size, and focus before recording and maintain parameters for all recordings – iris size should be adjusted to allow sufficient light for detection of the mouse body and surface disruption of the light path in the walkway. Field of view, frame rate (>60 frames per second, FPS), file type (.avi), and recording interface are controlled using StreamPix6 software. To ensure proper analysis of videos and reduce file size, the field of view should only comprise inside boundaries of the walkway.

6. Following setup, gently place the mouse at one end of the MouseWalker. Allow the mouse to acclimate to the walkway (>5 minutes), some mice may start walking immediately and others will need more time before starting to walk. Avoid excessive sounds and movement during recording to reduce experimental noise and allow mice to walk calmly.

7. Record a video of each mouse moving in a continuous line across the walkway. Eliminate recordings in which mice are running (all four paws are simultaneously out of contact with the walkway), mice do not complete the entire crossing, or unexpected waste (e.g., feces, urine) obscures the field of view. Four replicate videos are recorded for analysis of each mouse. Note, the direction of movement is not important for video analysis.

8. Save videos with an appropriate title for subject ID, experimental conditions, replicate number, and date.

9. Review the recorded videos for clarity and accuracy before completion of mouse recording, if additional videos are required, repeat from step 6.

10. Arrange videos from each experiment into corresponding folders for clarity and ease of access.

11. Proceed with video analysis.

#### B.3 Video Analysis with MouseWalker Software (Approximate Time: 30 minutes)

**Figure 2:**
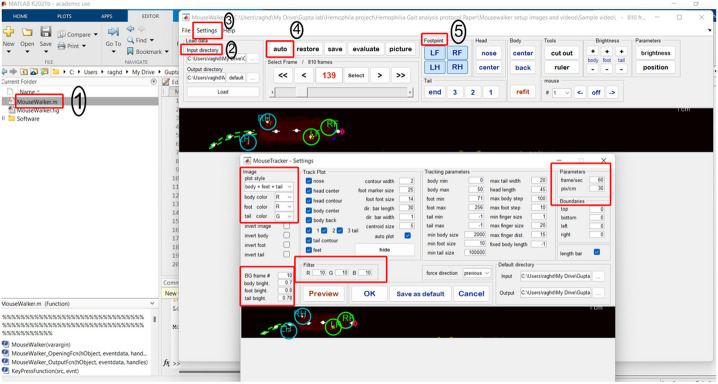
MouseWalker software setup and video analysis. (1) In MATLAB, run the MouseWalker file. (2) From the input directory, select the video folder to be analyzed, and in the output directory, select the desired location to save output data. (3) Select *“setting”* to adjust different parameters. First, set the frame rate, pixels/cm, plot style, and color and brightness. (4) After loading the video, press *“Auto”* to initiate automatic tracking to continue until the final frame. (5) Evaluate automatic detection of physical features frame-by-frame. Incorrect features can be removed and manually defined.

1. The MouseWalker software and documentation are open-source and available free of charge ^8^.

2. Download the necessary files and save them onto an appropriate, local hard disk.

3. Verify MATLAB installation and that the necessary plug-ins are ready. Run MATLAB.

4. In MATLAB, direct to the local address for the MouseWalker file and select *“Run”* to initiate the program interface (Figure 2).

5. It is critical to set calibration before importing and analyzing videos. Select *“Setting”* from the toolbar on the top right and input video parameters; these may vary depending on setup (e.g., working distance between camera and apparatus, workstation computational power, etc.).

6. First, set the frame rate; herein, we recorded at approximately 60 frames per second (FPS). Notably, higher frame rates may achieve greater video quality but require larger file storage.

7. Set the scale (pixels/cm) using the value acquired in the calibration procedure.

8. Other settings may be adjusted for robustness depending on size, age, strain, color, and other physical features of subjects. For reference, we include our optimized settings for the detection of average-sized (~25 g body weight) adults (>2 months), wild-type and hemophilia, and dark-furred mice.

9. From the input directory, select the video folder to be analyzed, ideally containing one video at a time.

10. From the output directory, select the desired location to save output data. The input folder may be selected to maintain consistent locations for raw video and analyzed data files.

11. Select “Load” and allow sufficient time for the video file to be opened by the software.

12. Select “Auto” to initiate automatic frame-by-frame detection of mouse features (I.e., mouse body, nose, tail, right forepaw (RF), left forepaw (LF), right hind paw (RH), left hind paw (LH), which are annotated in a video preview window.

13. Allow automatic tracking to continue until the final frame. Alternatively, if the mouse extends beyond the field of view before the video’s end, wait until the mouse’s nose reaches the end of the field of view, then select “Cancel”.

14. Following automatic tracking, return selection to the first video frame by manually typing the frame number or using arrow buttons. Evaluate automatic detection of physical features frame-by-frame. Incorrect features can be removed and manually defined. In the case of a misplaced right forepaw, select the “RF” button to undo the incorrectly identified feature, then select the “RF” button again, which will change the mouse pointer to a cursor symbol. With the cursor symbol, select the correct position for RF to place a new label.

15. After verifying the labeling is correct for all frames in which features are visible, select *“Evaluate”*. This will generate the analysis data and compile values into a Microsoft Excel file in the assigned output directory folder.

## Troubleshooting

Note: Delete generated analysis data from the output folder before reloading a video

1. Automatic tracking is oriented in the wrong direction

- After selecting *“Auto”*, the automatic tracking may falsely determine the direction in which the mouse is facing (e.g., labeling the nose as tail and vice-versa). There are two standard solutions. (1) Reload the video, after deleting any prior analysis, and skip past the first few frames (approximately 3-5 frames); the automatic tracking may properly orient and label the video from this point forward. (2) Open the *“Settings”* window and change the force direction selection, which is set to *“Previous”* by default, to the correct direction.

2. Multiple mice are shown as tracked

- Aberrations may appear as additional mice following automatic tracking. Select the corresponding *“Mouse Number”* for any falsely identified areas, then select *“Off”* to remove the inaccurate numbering.

3. Interference of reflection and fur

- Occasionally, automatic tracking may mistake portions of a subject’s body for paws. Use manual adjustment to properly unassign and reassign features. If fur color or reflections are a common occurrence, double-check light settings (i.e., brightness and color) and adjust accordingly.

4. Low signal from paws

Mouse fur color and body mass can vary greatly by strain, disease state, etc. Low body mass and small paw size may result in little signal intensity from fTIR. Polishing the acrylic surface with a small amount of mineral oil, or other non-reactive oil helps to increase refraction without increasing background and may be recommended for difficult acquisitions.

## Time Taken

### Anticipated Results

#### Gait analysis by MouseWalker:

One of the challenging steps in the gait analysis experiments is dealing with the analysis of the huge amount of generated data and interpreting the results. In this section, we provide a summary of the most important parameters as well as a guide to analyzing the data.

#### Gait cycle:

During walking, the gait cycle is usually subdivided into a stance phase and a swing phase. The swing phase is the period of time when the paw under consideration is not in contact with the floor. The stance phase is the period of time when the paw under consideration is in contact with the floor.

#### Interlimb coordination parameters:

- All-Stance Index refers to when all 4 paws are making contact, indicating a full stance.
- Single swing refers to a single paw in motion.
- Bound swing indicates simultaneous motion of both fore or hind limbs.
- Lateral swing indicates ipsilateral limb movement.
- Diagonal swing indicates contralateral limb movement.
- Three swing indicates that a single paw is making contact with the walkway.
- All swing indicates no paws are in contact.

**Figure 3:**
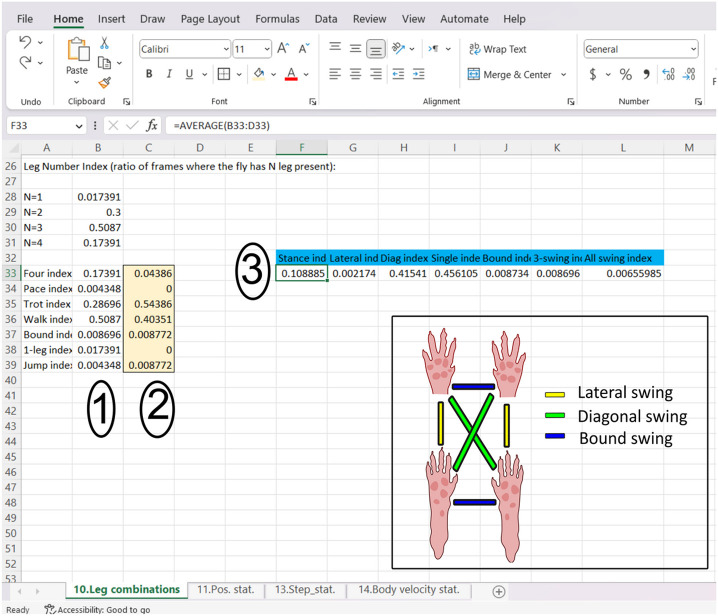
MouseWalker Data analysis This figure explains how to compile the data generated from video analysis. This is sheet 10, which demonstrates leg combinations, (1) is the data retrieved from one of the videos, and (2) is the data from a replicate video of the same mouse and the same parameters. (3) are the average values that will finally be used for that mouse. The inset shows the meaning of different leg combinations where Bound swing indicates simultaneous motion of both fore or hind limbs; lateral swing indicates ipsilateral limb movement, and diagonal swing indicates contralateral limb movement.

Interlimb combination data are unitless and calculated as a ratio of each instance over the total instance, and a large all-swing index may be indicative of running rather than walking behavior and may require follow-up assessment. The diagonal swing is the most representative leg combination, which increases with speed. The single swing index decreases significantly with increased speed. The all-legs swing index is observed primarily at higher speeds.^8^ The bound (front/hind), lateral swing may be used by the mouse to correct for present disturbances (Figure 3).

#### Positional parameters

- Anterior extreme position (AEP) is the relative distance of the paw from the center of the body upon contact.
- Posterior extreme position (PEP) is the relative distance at the end of the stance when the paw is lifted. AEP and PEP data are unitless and represent the distribution of relative positions of contact between limbs and the walkway and position when contact is broken, with larger values indicating lower stability and more variability in limb movement.
- Stance instability is calculated as the distance of deviation for limb location for each limb (in mm) and data are reported as a sum of all limbs.
- Body stability represents the sway of the subject’s body from a predicted straight line of motion (in mm)

**Spatiotemporal (step) parameters** relate to the position in space including step length, and speed of the swing, and to time including the duration of the stance phase, and the duration of the swing phase. Interpretation of these results is context dependent but may reveal individual limb dysfunction. Mice models with injury on one paw show shorter time on the ground (stance) which is compensated by a longer air time of that paw, reflected in a longer swing duration, and also associated with shorter step length.^9^

Two replicate videos are analyzed for each subject using the MouseWalker software, and data from the automatically generated Microsoft Excel spreadsheets are manually parsed to obtain averages from the replicates to analyze individual observations with appropriate statistical tests. For convenience, we provided an Excel sheet template (supplementary file 2), where the cells for the replicate video are highlighted orange and the averaged values are highlighted blue.

The calculated data used for post hoc analysis can be located in the auto-generated Excel file on sheets 10, 11, 13, and 14 (Figure 3).

**Sheet 10** contains data for leg combinations: these include (1) stance index (reported as “Four index”), (2) lateral index (reported as “Pace index”), (3) diagonal index (reported as “Trot index”), (4) single index (reported as “Walk index”), (5) bound index (reported as “Bound index”), (6) 3-swing index (reported as “1-leg index”), and (7) all swing index (reported as “Jump index”), which represent instances of contact between the walkway and (1) all limbs, (2) 2 ipsilateral limbs, (3) diagonal fore and hind limbs, (4) 3 limbs, (5) 2 contralateral fore or hind limbs, (6) single limb, and (7) no limbs, respectively.

**Sheet 11** contains positional data for limbs and reports anterior extreme position (AEP left-right diff) for forelimbs (cell B22) and hind limbs (cell D22), posterior extreme position (PEP left-right diff) for forelimbs (cell B23) and hind limbs (cell D23), stance instability for each limb, and body stability.

**Sheet 13** contains data for stance duration (s), swing duration (s), swing speed (cm/s), and step distance (cm) for each limb, which are then totaled for each parameter.

**Sheet 14** contains data for walking speed (mm/s) from individual frames, and these values are averaged at the bottom of the sheet (column B). Values should be checked against the literature for accuracy and may require calibration in the MouseWalker settings (Figure 2).

#### Weight Bearing using incapacitance measurement device:

**Figure 4:**
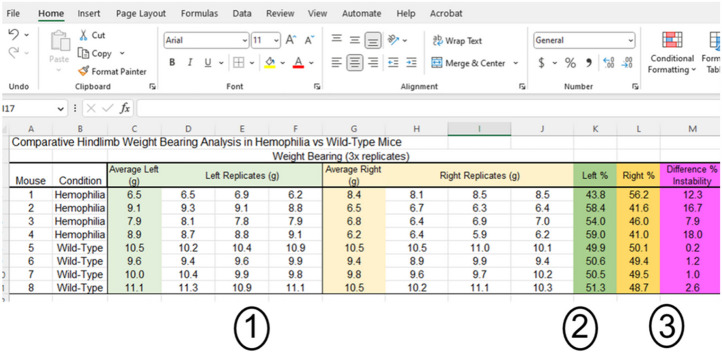
Weight Bearing Data analysis. This sample dataset indicates (1) replicate values for left and right hind paws from 3 separate evaluations of each subject (per row), which are then averaged for each hind paw. (2) The averaged weight bearing for each hind paw is then expressed as a percent of the total weight bearing for the left and right, separately. (3) Lastly, the absolute value of the difference between the percent of weight bearing is calculated and used for statistical comparisons. A greater value indicates greater instability.

The incapacitance weight-bearing device comprises two scales, one for each hindlimb, an enclosure to maintain mice in place on the scales, and a display that provides numerical data; these data may be exported via a USB 2.0 or newer flash storage device or manually recorded during acquisition. Weight-bearing data are displayed after each 5 s recording period on the incapacitance measurement device as mass (to the nearest 0.1 g) for each hindlimb. The 3 replicate values for each hindlimb are averaged. Averaged values for each hindlimb are then divided by the cumulative mass from both limbs to calculate the percent weight bearing for each hindlimb. Lastly, the absolute value of the difference between percent weight bearing for left and right hindlimbs is calculated, and this value (percent difference between hindlimbs) is reported as the stance instability for each subject (Figure 4). Greater values indicate a larger degree of stance instability, and may be an indication of poor motor function, joint dysfunction, arthropathy, and/or musculoskeletal pain.

#### Interpretation:

Pain plays a crucial role in survival, but uncontrollable chronic pain is a debilitating feature of many diseases. Pain evaluation suffers from poor quantitative methods, thus precluding effective pain management, however gait and weight bearing offer non-invasive, non-evoked quantitative measures of pain that can easily be translated into clinical settings from animal models. Gait and weight-bearing are intrinsically linked, and our approach using these complementary evaluation techniques may provide insight into arthropathy and the subtle motor alterations earlier which can help with the management of the disease. Our weight-bearing analysis provides a robust, numerical value to determine asymmetry or instability, especially in the hindlimb joints of our subjects, with greater instability associated with injury or disease progression. In support of these data, the MouseWalker assessment of gait provides detailed numerical outputs to describe sources of weight-bearing instability and motor dysfunction, for example, a disruption in leg combinations (leg indices) may indicate compensatory behavior arising from poor weight-bearing, or altered body stability may suggest further arthropathy or neurological deficits that other evaluations may be unable to detect.^10^
